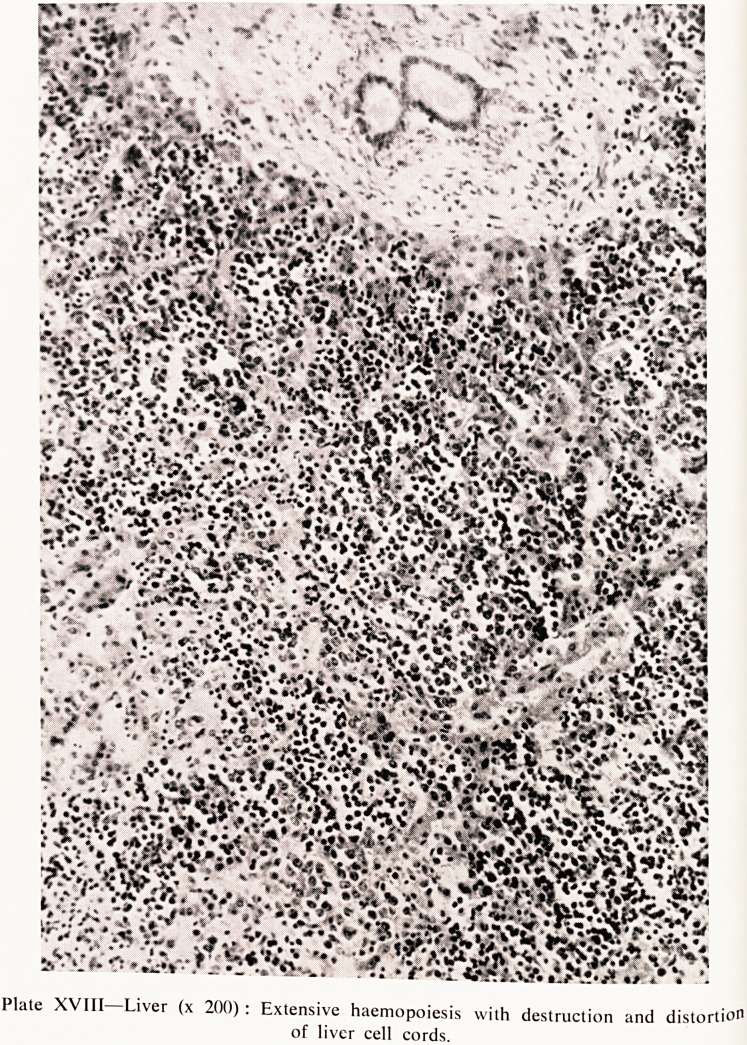# Haemolytic Disease of the Newborn with Pulmonary Atelectasis

**Published:** 1967-04

**Authors:** T. F. Hewer


### HAEMOLYTIC DISEASE OF THE NEWBORN WITH PULMONARY ATELECTASIS

*A Clinico-Pathological Conference held in the University of Bristol on 22nd November, 1965.*

Chairman: Professor T. F. Hewer

P.M. No. 9521

*Dr. Beryl Corner:* The mother of this baby had a very interesting obstetric history. She was a healthy woman, aged 33 years, group A, Rh negative. In 1955, her first baby was born at 37 weeks gestation and was healthy. In 1956 she had a macerated stillbirth at 39 weeks, weighing 4 lb. 8 oz. Fœtal death was thought to be due to hæmolytic disease of the newborn from rhesus incompatibility. Her third pregnancy in 1958 resulted in a stillbirth at 30 weeks gestation, weighing 1 lb. only, with gross hæmolytic disease. After this she had two normal babies by A.I.D. Dr. Tovey may tell us more about the blood groups of the mother and her husband, who was Rh (D) positive, homozygous.

The present pregnancy was by her husband. The Rh antibody titre at the beginning was 1/5, rising to 1/20 at 30 weeks, and at 34 weeks 1/40—1/80. The rising titre of antibodies and the history of two stillbirths due to hæmolytic disease suggested that action was necessary to get a live baby this time. Mr. John obtained samples of the amniotic fluid at 27, 30 and 34 weeks and the rising pigment levels to 0.097 in the third specimen suggested that the fœtus was moderately severely affected with hæmolytic disease. In view of the past history it was decided to deliver by elective Cæsarean section at 35 weeks.

The operation was performed uneventfully, and a female baby was obtained, weighing 5 lb. 5 oz. The child looked extremely pale and was very flaccid. She gasped at 2 minutes but intratracheal intubation and intermittent positive pressure oxygen was started at 3 minutes because respiration had not continued. The baby remained very pale and was sufficiently œdematous for us to use the term "hydropic". The liver was enlarged, the spleen was easily palpable and there was a good deal of ascitic fluid in the abdomen.

As soon as the intratracheal tube had been passed, 10 ml of ascitic fluid was removed to relieve pressure on the diaphragm. Regular respiration was established in about 20 minutes. The umbilical cord blood was investigated. The Hb was only 3.5 g%; as the normal level for a baby of this gestational age is about 16.5 to 17g%, this was an extremely low figure. The total bilirubin was 7.5 mg% (upper limit of normal: 3—3.5 mg%) so this was also more than double the normal level. These two findings, with a Coombs test which was strongly positive, and the blood group O Rh positive, suggested that this child was severely affected with Rh hæmolytic disease of the newborn.

The first problem was to keep the child alive because respiration was difficult to initiate and not well established. Two hours after birth she stopped breathing, and required resuscitation again. The second problem was to treat the hæmolytic disease. When respiration was re-established it was decided to do an exchange transfusion with group O Rh negative blood. This was performed rather slowly in view of the hydropic condition, but complete exchange (80 ml/lb body weight) was effected. At the end of the transfusion the plasma bilirubin was down to 5.1% which was a reasonable reduction.

Digoxin and antibiotics were given. ACTH was also given to stimulate cortisol production in the hope that we might get some effective suppression of the antibody reaction. The more serious problem was still the respiratory condition. The respiratory distress syndrome of the newborn, which affects approximately 14-15% of all immature infants, had developed rapidly. After delivery of an immature baby by elective Cæsarean section the risk of respiratory distress is very high indeed; it has been put at 70% in some centres and is at least 50% in our experience here.

The respiratory distress syndrome is characterised by grunting respirations and sucking-in of the intercostal spaces. Respiration usually becomes shallow and rapid, although in this case it remained slower than usual and there were many apnœic attacks. I should also have mentioned that at the time of birth the heart rate had dropped to 80/minute which indicated imminent cardiac failure, and the rate remained slow throughout, and was steadily falling in the last few hours. An attempt was made to treat the respiratory distress in the usual way, i.e., to maintain body temperature and oxygenation by incubator nursing in an increased atmospheric oxygen concentration. With such poor pulmonary ventilation these children develop both metabolic and respiratory acidosis which necessitates monitoring of the blood pH, $PCO_2$ and bicarbonate by the Astrup technique at frequent intervals. The metabolic acidosis was kept more or less under control by intravenous infusions of 8.4% bicarbonate in a 5% glucose drip, following the exchange transfusion. Finally in a last desperate effort we thought this baby's pulmonary ventilation might be helped by a respirator. The trachea was intubated and respiration was then assisted with a Starling pump. Respiration and metabolic acidosis improved as shown by the capillary blood pH 7.4 about 12 hours before death. Increasing œdema and ascites reminded us that we also had the persisting problem of severe hæmolytic disease. The child died at 46 hours with respiratory and cardiac failure which is the usual mode of death in these cases. Dr. Tovey will tell you about the hæmatological aspects of the case.

*Professor Hewer:* I think you said, Dr. Corner, that this baby was conceived by artificial insemination?

*Dr. Corner:* Not this baby. This woman had lost two babies and then had two normal children by artificial insemination by donor. This one was by her own husband again.

*Professor Hewer:* Would Dr. Tovey like to say anything now?

*Dr. G. H. Tovey:* Perhaps it would be appropriate for me to outline the investigations which are undertaken to decide how severely affected the baby is *in utero*. The most important of these is the amniotic fluid examination. This determines how much bile pigment is present in the amniotic fluid and indicates the extent to which the baby's red cells are being hæmolysed by the mother's antibodies.

The result is obtained by spectrophotometric analysis and is then plotted on graph paper, since a decision regarding the normal level is related to the period of gestation. An amount of pigment which would be normal at 32 weeks would be pathological at 36 weeks because of the greater maturity. The graph is therefore divided into three zones and one can say that if the amount of pigment lies in the lower zone the fœtus is only mildly affected or may be Rhesus negative. If, by contrast, the amount of pigment lies in the upper zone the mother is carrying a severely affected baby.

We have now followed up nearly 200 of these cases and have obtained almost complete accuracy of prediction in both the upper and lower zones, but only about 75% reliability when there is a mid-zone value, which was unfortunately the result obtained in this particular analysis. We now know, in fact, that even though only a moderate amount of pigment is present in the amniotic fluid there is a 1 in 30 chance that the woman is carrying a baby so severely affected that it will die *in utero*, or shortly after delivery despite exchange transfusion. With the advantage of greater experience, if this patient were carrying this baby to-day, we should recommend an intrauterine transfusion despite only a moderate amount of pigment being present. Twelve months ago the technique of transfusing the foetus *in utero* was rightly regarded as hazardous both to the mother and the baby, and since the mother had three live children, one by her husband and two by A.I.D., we decided that the additional hazard did not justify intrauterine transfusion. During the past 12 months improvements in techniques have eliminated most of the hazards, so that despite the pigment being in the mid-zone, the history and antibody titre are such that this baby would to-day be given an intrauterine transfusion.

*Professor C. Bruce Perry:* Perhaps Dr. Tovey would explain the Liverpool technique of preventing this altogether when you know it is likely to happen.

*Dr. Tovey:* This is an exciting but headache-provoking development, because it now looks as though it may soon be possible to prevent Rh negative mothers from forming Rhesus antibodies. I will tell you shortly where the headache comes in! About five years ago a research group in Liverpool started to consider why only some Rh negative women carrying Rh positive babies form Rh antibodies. They thought it probable that antibodies would form only if there was a leak of the baby's red cells into the maternal circulation, and that the most likely time for this to happen would be during labour, when the placental barrier would be under maximal stress. Their first investigation was therefore to see whether fœtal leaks occur only during labour. A relatively simple technique was used in which films of the mother's blood are flooded with a citric acid phosphate buffer at pH 3.4. This solution removes adult hæmoglobin from the red cells but not fœtal hæmoglobin so that when the treated film is then stained and examined microscopically, the only cells which will stain in the normal manner are those which have leaked into the mother's circulation from her baby. By this technique it was established that approximately 90% of fœtal leaks occur during labour. The Liverpool team next wondered whether the formation of Rh antibodies required not only a fœtal leak, but also that the Rh positive foetal red cells must remain in the mother's blood for at least 48 hours before she could become immunised to the Rh factor. They therefore enrolled a panel of male Rhesus negative donors who were willing to be injected with Rhesus positive blood and some 24 hours after this injection they were given an intravenous injection of high titre Rhesus antibodies. The object of the injection of the Rhesus antibodies was to destroy the previously injected Rh positive red cells. After several months of experimentation it was found that none of the males who had received injections of both Rh positive red cells and anti-Rhesus serum developed any Rhesus antibodies, whereas by contrast, 50 per cent of a control series who

Plate XVII—Lung (x 80) : Atelectasis with hyaline membrane.

Plate XVIII—Liver (x 200): Extensive haemopoiesis with destruction and distortion of liver cell cords.

did not receive an anti-Rhesus injection, or who were given it more than 48 hours after the red cells were injected, subsequently became immunised to the Rh antigen and developed Rh antibodies.

Following this success, the next step was to persuade a group of Rhesus negative primiparous mothers to be tested for a fœtal leak shortly after delivery, and of those in whom there was a leak of not less than 0.5 ml of the baby's blood, one half were given an injection of anti-Rhesus within 36 hours of delivery, whilst the remainder acted as controls. In this trial 78 Rh negative mothers, all of whom had had a significant leak of fœtal red cells, have been given an anti-Rhesus injection in the form of gamma globulin concentrate. This has two advantages; firstly the injection can be given intramuscularly, and secondly there is no risk of transmitting hepatitis. Of the 78 mothers given gamma globulin, none had formed any Rhesus antibodies up to six months after delivery. Of the 78 controls given no anti-D gamma globulin 19, or nearly 25 per cent, subsequently formed Rhesus antibodies.

This work has now been extended to New York, Germany and California, and in all four centres the results are essentially the same (Clarke *et al.*, 1966). There is, however, likely to be a delay of another few months before it is established whether immunisation to the Rh factor can be prevented by this means, because the real test will come when some of the women given gamma globulin are pregnant with another Rhesus positive baby. Will they be fully protected, or will the protection only be short-lived so that they quickly respond by forming antibodies? To date some 18 of these women have become pregnant again, and as yet none has formed Rhesus antibodies. It looks therefore as if all this is working out and I think that within the next six months we are likely to see increasing demands for injections of gamma globulin containing anti-D. This is what I was referring to earlier as a "headache-provoking development"!

The big problem will be collection and preparation of all the gamma globulin required, since it will have to come principally from women like this patient who already possess strong Rh antibodies. On estimate, to protect all the Rhesus negative primiparous mothers in the South West Region we shall require a panel of 60-70 women with a high titre of Rhesus antibody, all of whom are prepared to give one litre of blood at least six times a year to provide the 350-400 litres of blood likely to be required.

*Dr. O. C. Lloyd:* You will have to use men!

*Dr. Tovey:* I was in Holland recently. There they have started recruiting a panel of women, with, so far, a very good response.

*Professor Hewer:* Thank you very much. If there are no questions I will ask Dr. Andrews for the post mortem findings.

*Dr. Andrews:* At post mortem this was an extremely pale, slightly jaundiced infant with well marked œdema of the legs and trunk and also slight oedema of the face, causing stretching of the overlying skin, which appeared tight and rather shiny.

Internally there were signs of anoxia with petechial hæmorrhage over the surface of the heart which was otherwise normal, and also over the surface of the lungs which were almost completely unexpanded except for a narrow rim of tissue around the edges. On histological examination the lungs showed expansion down to terminal bronchioles and alveolar ducts with atelectasis beyond, and patchy interstitial hæmorrhage (Plate XVII).

The liver was enlarged and congested and weighted 147 g. The normal for this period of gestation would be around 100 g. so that the liver is about 1½ times normal size. Histology of the liver showed extensive extra-medullary hæmopoiesis (Plate XVIII). The cords of liver cells were distorted and destroyed by hæmopoietic tissue in which many immature blood cells were present, mostly of the erythroblast series. Only a small amount of surviving liver tissue could be identified. When the liver was stained for iron by the Prussian Blue reaction quite a lot was found to be present, both in hepatic parenchymal cells and in reticulo-endothelial cells. The presence of iron is, of course, to be expected in any severe hæmolytic process such as this infant suffered from.

The spleen was also enlarged, weighing 16 g. while the normal for this age would be about 6 or 7 g., so the spleen was just over twice the normal size. It was very dark and quite firm. Histology showed a marked diminution or even absence of the lymphoid follicles so that quite often the so-called central artery was surrounded by red pulp without any lymphocytes at all. The red pulp was congested and contained large numbers of immature cells.

No other organs apart from the liver and spleen showed any persistence of extra-medullary hæmopoiesis. I say persistence because such hæmopoiesis usually ceases at birth even in cases like this one.

The placenta weighed 630 g. which was nearly one third of the weight of the infant, while 1/5 to 1/6 at 35 weeks would be nearer normal. It was swollen and pale with a deeply fissured surface. Histological examination showed œdema of villi with many nucleated red cells in the blood vessels. Persistence of Langhans layer was not found.

The brain showed congestion of superficial vessels and numerous pin-point hæmorrhages on the cut surface, due to anoxia. There was no kernicterus and of course the bilirubin level was never high enough to cause this.

In summary then, this is an infant with hæmolytic disease of the newborn dying from the effects of profound anæmia and associated pulmonary atelectasis.

*Dr. Corner:* Could I ask you, in the section of lung was there any hyaline membrane in the alveolar ducts?

*Dr. Andrews:* Yes, there were some scattered patches of hyaline material lining alveolar ducts and bronchioles. It was rather irregular in distribution but I think it was early hyaline membrane.

*Professor Hewer:* Did the baby make great efforts to breathe?

*Dr. Corner:* Oh yes. In the intervals between the apnœic attacks great efforts were made. The chest was overdistended antero-posteriorly and sucked-in laterally. The respiration rate was not as fast as we sometimes see in these babies but it was raised to 50-60 per minute at times.

*Dr. N. J. Brown:* Did the baby have any pleural effusions?

*Dr. Andrews:* Yes. She did have small bilateral effusions. About 15 ml. on each side.

*Professor Perry:* Could Dr. Brown tell us why he asked that question?

*Dr. Brown:* Because, sir, I think that one of the main reasons why they don't breathe is that there is collapse of the lungs due to the presence of pleural effusions.

*Professor Hewer:* Compression collapse.

*Dr. Brown:* Yes. Coupled with ascites which pushes the diaphragm up.

*Professor Hewer:* There was ascites, I take it.

*Dr. Corner:* Yes. We have been very unlucky with these hydropic babies. We have not kept alive any baby who is obviously hydropic. A few who have been a little œdematous have lived, as well as one or two severely anæmic babies which have been treated with immediate exchange transfusion.

*Professor Hewer:* What is the prospect of subsequently recognised cerebral damage if you do this?

*Dr. Corner:* Well, cerebral damage depends firstly on whether the infant becomes severely jaundiced, which most babies with hydrops fœtalis do not, and the other damaging factor is apnœic attacks resulting in severe hypoxia. We are trying to correct the effects of hypoxia by biochemical control and correction of the metabolic acidosis which causes cerebral œdema. This particular baby had small cerebral hæmorrhages due to hypoxia which, if it had survived, might have caused clinical abnormality.

*Dr. D. W. Barritt:* Was there any rise in venous pressure?

*Dr. Corner:* Pressure in the umbilical vein was up to 17 cm. and was reduced to 12.5 cm.

*Dr. Barritt:* Did you do this by drugs, do you mean?

*Dr. Corner:* No, by taking off blood.

*Professor Perry:* Is the anasarca due to heart failure and severe anaemia?

*Dr. Tovey:* Yes, it is considered to be.

*Professor Perry:* I was a little worried when I learned that to correct the acidosis you were giving them bicarbonate, I would have thought this wouldn't have helped the heart failure. You're between the Devil and the Deep Blue Sea!

*Professor Hewer:* You do a venesection when you gradually replace the infant's blood with Rhesus negative blood?

*Dr. Corner:* When the umbilical vein is catheterised for the transfusion the venous pressure is taken and blood is removed to reduce the pressure if necessary. Dr. Cain is here, and he actually treated this baby.

*Dr. A. R. R. Cain:* Yes. One of the problems was to decrease the venous pressure; I do not think I was very successful because to bring it down to normal (5-6 cm.) would have required removal of about 50-60 ml. of blood and the total blood volume of this baby was only of the order of 200 ml.

*Dr. Corner:* It has been said that the prognosis for the respiratory distress syndrome is better if the venous pressure is raised than if it is normal or low. So the pressure in this case might have helped the pulmonary condition.

*Dr. Ray:* Dr. Corner, is there any place for a ventilator in the treatment?

*Dr. Corner:* There is a big technical problem here. In several centres trials are being done on comparison of treatment by routine methods or assisted respiration with a patient-triggered ventilator. I think it is too soon to say whether the results are better with the ventilator. A gastrostomy is sometimes done also to feed directly into the stomach and thus prevent gastric distension which adds to respiratory difficulty.

*Dr. Lloyd:* This is to help the poor ventilation of the respiratory distress syndrome?

*Dr. Corner:* Yes.

*Dr. Lloyd:* And is not for the erythroblastosis?

*Dr. Corner:* No, but these hydropic babies are nearly always immature because to prevent more severe fœtal damage early delivery is always carried out either by induction of labour or Cæsarean section. Therefore even if we can cope with erythroblastosis successfully there is still the risk of respiratory problems. The immaturity always create a double problem.

*Mr. Keen:* I wonder if the fundamental pulmonary problem in a hydropic infant is akin to that of the respiratory distress syndrome of the newborn, namely considerable stiffening of the lungs, or put more physiologically, a decrease in compliance, and that if this is so, death is due to hypo-ventilation. Could Dr. Corner tell us whether or not intubation and artificial ventilation has been used in these patients and if so with what effect?

*Dr. Corner:* Yes, there is no doubt that we do not always tackle these problems energetically enough. Everything depends on the first ten minutes of the baby's life. Unless you have all facilities mobilized, vital time is lost and an irreversible condition develops. If we anticipated another bad case we ought to make more elaborate preparations for immediate action with artificial ventilation if necessary.

*Dr. Lloyd:* When you get cases of respiratory distress of the newborn which sorts can you be fairly sure are going to do well, as opposed to the bad ones?

*Dr. Corner:* Well, there are certain clinical signs which guide us as to how the baby is doing. Probably the most important of all is the maturity of the baby. The more immature the worse the prognosis. Respiratory rate is another, and the degree of intercostal and sternal recession. If in very small babies this is very gross then there is obviously not much lung expansion. Auscultation of the lung is quite helpful. If they are full of crepitations with not much air entry at an early stage it indicates that there is severe pulmonary œdema. To-day all these patients have 4-hourly estimations of blood pH, bicarbonate and $pCO_2$. If $pCO_2$ remains high these are the most difficult cases to treat because $pCO_2$ can only be effectively lowered by improved oxygen exchange, and pulmonary œdema prevents this. Unfortunately we have no means of artificial blood oxygenation. We really should do blood oxygen estimations, i.e., $pO_2$, because this would be our best guide as to the efficiency of pulmonary gaseous exchange, but the technical problems are considerable. Some babies are probably not getting a high enough oxygen concentration in the inspired air.

*Professor Hewer:* May I ask, Dr. Corner, how many of these babies have in fact survived after this treatment? Are there enough for you to say what the prognosis is going to be? I should have thought that it is a most hazardous business.

*Dr. Corner:* Bergerot-Blondel and Troisier (1964) in Paris reported eight survivors in a series of 19 cases of severe hydrops fœtalis using specialised treatment techniques. These survivors all appeared to be normal children at 5 to 6 year old. We have no surviving cases of severe hydrops fœtalis, although at least three severely anæmic babies (Hb 25-35% in cord blood) have lived and are normal at follow-up.

*Student:* May I ask Dr. Tovey a question: I think it is true to say that the work at Liverpool started by someone noticing that when the child was incompatible with the mother in the ABO system the mother did not seem to develop Rhesus antibodies. Is there therefore any method by which you can forecast whether or not the child will be compatible?

*Dr. Tovey:* No, but fortunately that is not necessary. The mother will not be given the anti-D gamma-globulin until after delivery, otherwise the anti-D may harm the baby. If the baby is Rh negative the mother will not need an injection and, until supplies of gamma-globulin are adequate, the mother who has given birth to an ABO incompatible Rh positive baby is unlikely to get an injection because there is only a slight risk that she will develop Rh antibodies. Prophylactic injections will be limited therefore to those Rh negative primiparæ who give birth to a Rhesus positive baby, preferably when tests have been done to detect fœtal blood in the mother's circulation and when the baby's ABO group is found to be compatible with the mother's.

REFERENCES

Bergerot-Blondel, Y. and Troisier, S. (1964), Communication to Xth General Assembly, Medical Women's International Association.

Clarke, C. A. *et al.* (1966), *Brit. med. J.*, **ii**,,907-914.

## Figures and Tables

**Plate XVII f1:**
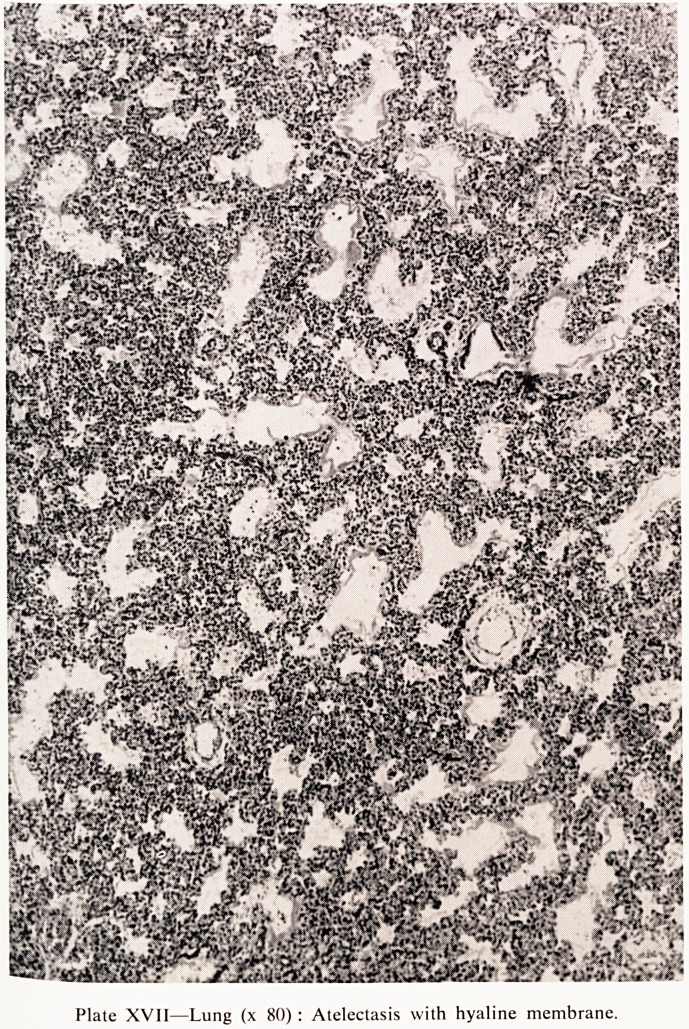


**Plate XVIII f2:**